# Hysteretic Dynamics of Multi-Stable Early Afterdepolarisations with Repolarisation Reserve Attenuation: A Potential Dynamical Mechanism for Cardiac Arrhythmias

**DOI:** 10.1038/s41598-017-11355-1

**Published:** 2017-09-07

**Authors:** Kunichika Tsumoto, Yasutaka Kurata, Kazuharu Furutani, Yoshihisa Kurachi

**Affiliations:** 1 Department of Pharmacology, Graduate school of Medicine, Osaka University, Suita, 565-0871 Japan; 2 Department of Physiology, Kanazawa Medical University, Ishikawa, 920-0293 Japan; 3 Global Center for Medical Engineering and Informatics, Osaka University, Suita, 565-0871 Japan; 4 Department of Physiology and Membrane Biology, University of California Davis, Davis, 95616 USA

## Abstract

Some cardiovascular and non-cardiovascular drugs frequently cause excessive prolongation of the cardiac action potential (AP) and lead to the development of early afterdepolarisations (EADs), which trigger lethal ventricular arrhythmias. Combining computer simulations in APs with numerical calculations based on dynamical system theory, we investigated stability changes of APs observed in a paced human ventricular myocyte model by decreasing and/or increasing the rapid (*I*_Kr_) and slow (*I*_Ks_) components of delayed rectifying K^+^ current. Upon reducing *I*_Kr_, the APs without EADs (no-EAD response) showed gradual prolongation of AP duration (APD), and were annihilated without AP configuration changes due to the occurrence of saddle-node bifurcations. This annihilation caused a transition to an AP with EADs as a new stable steady state. Furthermore, reducing repolarisation currents (repolarisation reserve attenuation) evoked multi-stable states consisting of APs with different APDs, and caused multiple hysteretic dynamics. Depending on initial ion circumstances within ventricular myocytes, these multi-stable AP states might increase the local/global heterogeneity of AP repolarisations in the ventricle. Thus, the EAD-induced arrhythmias with repolarisation reserve attenuation might be attributed to the APD variability caused by multi-stability in cardiac AP dynamics.

## Introduction

Early afterdepolarisation (EAD)^1^, which is believed to trigger lethal arrhythmias such as *torsade de pointes* (TdP) in patients with long QT syndromes (LQTS)^2, 3^ and heart failure^4^, is a repolarisation abnormality in the cardiac action potential (AP). In cardiomyocytes, outward ionic currents that play a critical role in AP repolarisation are comprised of multiple components, including the slow (*I*_Ks_) and rapid components (*I*_Kr_) of the delayed-rectifier K^+^ channel currents, the inward rectifying K^+^ channel current (*I*_K1_), and Na^+^-K^+^ pump (NaK) current (*I*_NaK_). Although these multiple components for repolarisation appear somewhat redundant when viewing cardiomyocytes as a system, this redundancy confers robustness to AP repolarization; should diseases or drugs diminish any one of the outward currents (e.g., *I*_Ks_ or *I*_Kr_), the others form an available reserve for AP repolarisation. This concept is referred to as the repolarisation reserve^5, 6^. Thus, a fundamental understanding of the relationship between the repolarisation reserve and EAD is essential if we are to gain new insights into the prevention of lethal arrhythmias, including drug-induced arrhythmias^7^.

Numerous experimental^8–12^ and theoretical studies^13–15^ have shown that attenuating the repolarisation reserve caused the prolongation of AP duration (APD) and subsequent EAD development. Although it is believed that excessive APD prolongation produces EAD via destabilisation of the membrane potential (*V*_m_)^10, 16^, the effects of EAD generation on configuration changes in AP remain unclear. Moreover, a recently published study^17^ demonstrated that pharmacological inhibition of *I*_Kr_ in rabbit ventricular myocytes (VMs) caused spontaneous and intermittent switching behaviour in the APs with and without EADs. Thus, the dynamical mechanism of EAD formation resulting from disruptions in the repolarisation reserve might be more complex than currently believed^18, 19^.

In the present study, we investigated dynamical stability changes of APs observed in a paced human VM model with *I*_Kr_ and *I*_Ks_ manipulation by combining computer simulations of APs with numerical calculations based on dynamical system theory, i.e. bifurcation theory^20, 21^. We show that EAD formation is caused not by *V*_m_ destabilization resulting from the excessive prolongation of APs without EADs (no-EAD response), but by abrupt transition to a different steady state via a bifurcation due to the reduction of *I*_Kr_ and/or *I*_Ks_. Our findings would provide a theoretical background for the prevention and treatment of EAD-induced arrhythmias in patients with LQTS and heart failure, as well as drug-induced arrhythmias.

## Results

### Hysteretic dynamics of AP responses in the VM model

**Figure 1:**
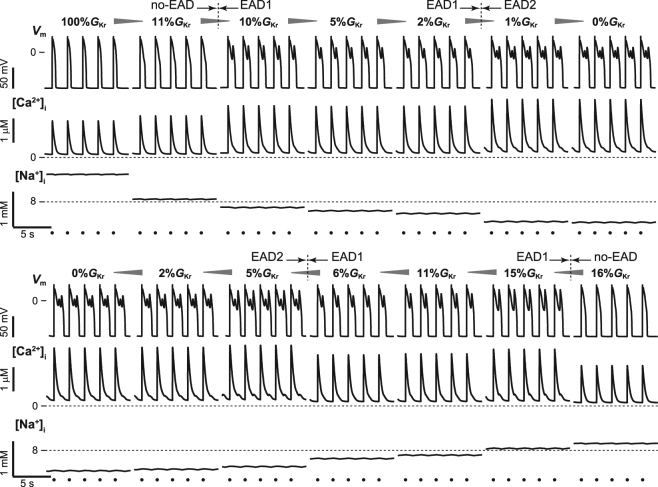
Simulated hysteretic switching dynamics of early afterdepolarisations (EADs) in the ventricular myocyte model during *I*_Kr_ manipulation. Steady-state action potential (AP) trains (top row), intracellular Ca^2+^ concentration, [Ca^2+^]_i_, (middle row), and intracellular Na^+^ concentration, [Na^+^]_i_, (bottom row) in each %*G*_Kr_. Each steady-state AP response was calculated by setting the sampled state variable values obtained from the steady-state AP response for the preceding %*G*_Kr_ as an initial condition. The dots represent the timing of applied current pulses. Pacing cycle length = 2 s.

Figure 1 shows steady-state AP trains observed in the paced VM model when the *I*_Kr_ conductance (%*G*_Kr_), which was expressed as a percentage of the maximal conductance value of *I*_Kr_ (see Methods), was reduced or increased. First, %*G*_Kr_ was set to 100% as the control condition, and the steady-state AP response was acquired. To investigate the effects of repolarisation reserve attenuation on AP response, %*G*_Kr_ was gradually reduced in 1% intervals. For instance, when 1% was reduced from the control condition of %*G*_Kr_ (i.e., 99%*G*_Kr_), the steady-state AP response was acquired by starting with the steady-state variable values of the AP response obtained just before the reduction of %*G*_Kr_ (i.e., 100%*G*_Kr_; Supplementary Table S1) as an initial condition. The reduction of %*G*_Kr_ until 11% caused AP prolongation in the no-EAD response, elevation of transient peaks and diastolic intracellular Ca^2+^ concentration ([Ca^2+^]_i_), and decline in intracellular Na^+^ concentration ([Na^+^]_i_) (Fig. 1, top). Subsequently, a slight reduction of %*G*_Kr_ to 10% caused the no-EAD response to transform into AP with EAD, which in turn caused a single small depolarised potential during AP phase 2 (EAD1), where the transient [Ca^2+^]_i_ peaks markedly increased and [Na^+^]_i_ further decreased. Such a transition between different AP responses was also elicited between 2%*G*_Kr_ and 1%*G*_Kr_; the EAD1 response transformed to an EAD2 response, characterised by two small depolarisations during AP phase 2.

In contrast, when %*G*_Kr_ was increased from 0%*G*_Kr_ at which EAD2 response was observed, a transition from EAD2 to EAD1 occurred between 5%*G*_Kr_ and 6%*G*_Kr_ (Fig. 1, bottom). With increasing %*G*_Kr_ from 5% to 6%, the diastolic [Ca^2+^]_i_ and transient [Ca^2+^]_i_ peaks declined while [Na^+^]_i_ elevated. Further increasing %*G*_Kr_ to 16% caused the disappearance of the EAD1 response, resulting in a transition to the no-EAD response. Of note, two distinct AP responses appeared at the same %*G*_Kr_ value (refer to 11%, 5%, and 2%*G*_Kr_ in Fig. 1). Such responses are known as a bi-stable phenomenon in which both of two distinct APs appears with the same parameter set, depending on an initial condition. Furthermore, the transitions between distinct AP responses observed in the decrease and increase of %*G*_Kr_ exhibited hysteretic characteristics. This suggests that the AP state was highly dependent on ion concentrations (especially in [Na^+^]_i_) within the VM just prior to the manipulation of %*G*_Kr_.

### Effects of changes in repolarisation reserve on dynamical stabilities of AP responses

**Figure 2:**
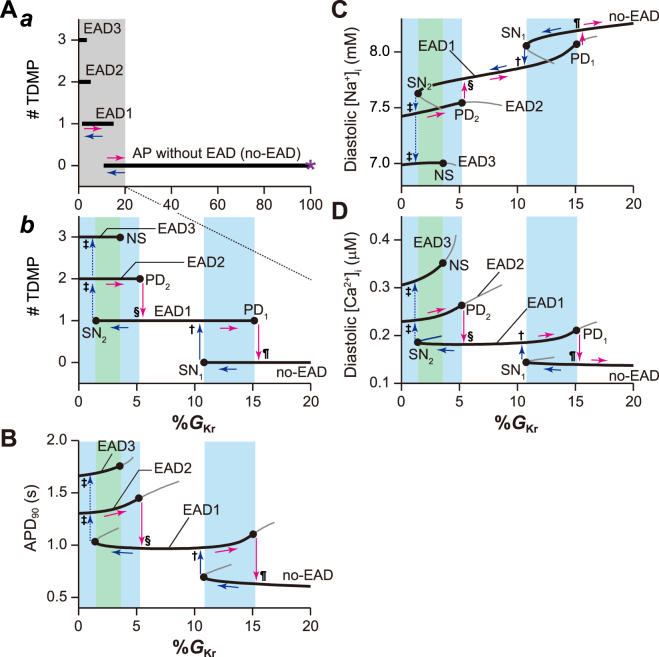
Effects of decreasing *I*_Kr_ on early afterdepolarisation (EAD) formation. One-parameter bifurcation diagrams of the number of transiently depolarised membrane potentials (#TDMP) during (**A**) action potential (AP) phase 2–3, (**B**) AP duration (APD) measured at 90% repolarisation, APD_90_, (**C**) diastolic [Na^+^]_i_, and (**D**) diastolic [Ca^2+^]_i_, as a function of the maximum conductance (%*G*_Kr_) of *I*_Kr_. In A, the panel *b* shows an enlargement of the grey region of %*G*_Kr_ within the panel *a*. Thick solid and thin grey lines represent parameter values at which stable and unstable periodic AP responses can be observed in the paced ventricular myocyte model, respectively. The %*G*_Kr_ ranges shown by cyan and green shading indicate parameter ranges at which the ventricular myocyte model exhibits bi- and tri-stable AP dynamics, respectively. SN, saddle-node bifurcation; PD, period-doubling bifurcation; NS, Neimark-Sacker bifurcation.

Next, we investigated the effects of attenuating and enhancing the repolarisation reserve on the dynamical stabilities of AP. Figure 2 shows one-parameter bifurcation diagrams for each AP with 100%*G*_Ks_ as a function of %*G*_Kr_. When *I*_Kr_ was at the control condition, i.e. 100%*G*_Kr_ (asterisk in Fig. 2A*a*), a normal no-EAD response existed uniquely as a steady-state AP response. As %*G*_Kr_ decreased (see horizontal blue arrows in each panel of Fig. 2), the no-EAD response prolonged the APD measured at 90% repolarisation (APD_90_) (Fig. 2B), while diastolic [Na^+^]_i_ (Fig. 2C) and [Ca^2+^]_i_ (Fig. 2D) declined and increased, respectively. The occurrence of saddle-node (SN) bifurcation at 10.8%*G*_Kr_ (SN_1_) led to the annihilation of the no-EAD response. Immediately after the disappearance of the no-EAD response, EAD1 (as in Fig. 1) manifested as another stable periodic oscillation (vertical blue arrows annotated with a dagger in each panel of Fig. 2). Then, the number of transiently depolarised membrane potentials during AP phase 2–3 (#TDMP) was increased by one (Fig. 2Ab). At the same time, the transition to EAD1 caused the abrupt prolongation of APD, a marked decline in diastolic [Na^+^]_i_, and an increase in diastolic [Ca^2+^]_i_ (Fig. 2B–D). As %*G*_Kr_ further reduced, EAD1 disappeared together with an unstable solution due to the occurrence of another SN bifurcation at 1.43%*G*_Kr_ (SN_2_), causing the transition to EAD2 (see vertical blue arrows annotated with double daggers in each panel of Fig. 2). This transition also led to further AP prolongation, decreased diastolic [Na^+^]_i_ and increased diastolic [Ca^2+^]_i_. Notably, bifurcation analysis found that EAD3, a third type of AP response characterised by three small depolarisations during AP phase 2, coexisted with the EAD2 response. Whether EAD2 or EAD3 was elicited depended on the initial conditions, such as [Na^+^]_i_ and [Ca^2+^]_i_.

In contrast, when %*G*_Kr_ was increased (horizontal magenta arrows in each panel of Fig. 2) from a condition of *I*_Kr_ loss (0%*G*_Kr_), the EAD2 response was destabilised via a period-doubling (PD) bifurcation at 5.2%*G*_Kr_ (PD_2_). This caused a transition to the EAD1 (see vertical magenta arrows annotated with section signs in each panel of Fig. 2). Further increasing %*G*_Kr_ led to the EAD1 destabilisation due to the occurrence of a PD bifurcation at 15.08%*G*_Kr_ (PD_1_), causing a transition to a no-EAD response (pilcrows in each panel of Fig. 2). Thus, transitions between distinct AP responses were accompanied by jumps in APD_90_ and in [Na^+^]_i_ and [Ca^2+^]_i_ (vertical blue and magenta arrows). Furthermore, changes in dynamical stabilities of AP responses when %*G*_Kr_ was reduced (horizontal blue arrows) or increased (horizontal magenta arrows) created hysteretic loops.

### Ionic mechanism of bi-stable dynamics and EAD formation

**Figure 3:**
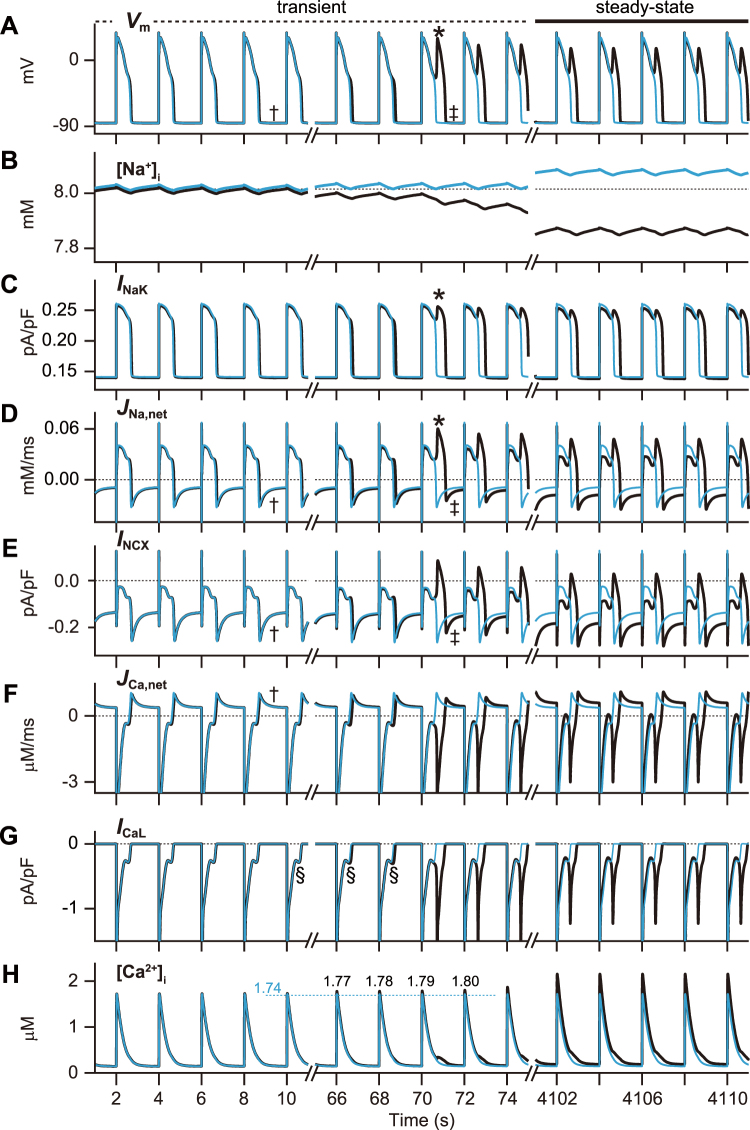
Ionic mechanism of the bi-stable dynamics between action potentials (APs) without and with early afterdepolarisation (EAD). Simulated APs and the changes in (**A**) membrane potential (*V*_m_), (**B**) [Na^+^]_i_, (**C**) *I*_NaK_, (**D**) net Na^+^ flux, *J*_Na,net_, (**E**) *I*_NCX_, (**F**) net Ca^2+^ flux, *J*_Ca,net_, (**G**) *I*_CaL_, and (**H**) [Ca^2+^]_i_. Each simulation was started from initial values of [Na^+^]_i_ = 8.03 mM (cyan traces) or [Na^+^]_i_ = 8.02 mM (black traces). Other initial values were set at the state variable values obtained from the AP without EAD (no-EAD) at 12%*G*_Kr_. In panel H, the numerical values indicate the peak [Ca^2+^]_i_ values (μM) when the AP dynamics in the ventricular myocyte model converges to AP with (black) and without (cyan) EAD, respectively.

Each corresponding AP response could be observed, depending on initial conditions, in the %*G*_Kr_ ranges at which each solid black line overlapped in Fig. 2. We referred to those observations as bi-stable and/or multi-stable phenomena. Next, we investigated how the same %*G*_Kr_ elicited bi-stable AP dynamics without and with EAD. Figure 3 shows each AP profile, [Na^+^]_i_, [Ca^2+^]_i_ and ion currents associated with their ion concentration variabilities. Initial values, except for [Na^+^]_i_, were set to the same values at which the VM model exhibited the steady-state no-EAD response at 12%*G*_Kr_ (Supplementary Table S2). When [Na^+^]_i_ was set to a value (8.03 mM) near the state transition threshold, evoked APs converged to no-EAD responses (cyan trace in Fig. 3A), and [Na^+^]_i_ increased due to a gradual accumulation of Na^+^ (cyan trace in Fig. 3B). This elevated [Na^+^]_i_ enhanced NaK activity, which resulted in relatively large *I*_NaK_ (Fig. 3C) via an increase in Na^+^ extrusion (Fig. 3D) during AP phase 2. Therefore, the outward current during the AP phase 2 was sufficient for the completion of AP repolarisation. In contrast, when [Na^+^]_i_ was set to 8.02 mM (black trace in Fig. 3A), the evoked APs were gradually prolonged with each stimulation, and eventually converged to an EAD1 response, which caused a marked decline in [Na^+^]_i_ (black trace in Fig. 3B). A prolongation of the active duration of *I*_NaK_ due to AP prolongation increases Na^+^ extrusion. In particular, emerging EAD (asterisk in Fig. 3A) markedly increased Na^+^ extrusion (asterisk in Fig. 3D), consequently leading to a reduction in [Na^+^]_i_ (Fig. 3B). On one hand, the Na^+^/Ca^2+^ exchanger (NCX) current, *I*_NCX_ (Fig. 3E), contributed to increases in [Na^+^]_i_ via Ca^2+^ extrusion and Na^+^ loading, particularly during the diastolic phase (daggers in Fig. 3D and F). However, increased [Na^+^]_i_ was restricted because the diastolic interval (DI) was shortened by AP prolongation due to emerging EAD (see double daggers in Fig. 3A,D and F). Eventually, [Na^+^]_i_ in the EAD1 response converged to a lower value than that in the no-EAD response (black traces in Fig. 3B). This lowered [Na^+^]_i_ diminished NaK activity, thereby causing *I*_NaK_ to decrease during AP phase 2 (Fig. 3C), and accelerating AP prolongation.

The AP prolongation mediated by the [Na^+^]_i_ decline slowed AP repolarisation (Fig. 3A and Supplementary Fig. S1A*a*), which delayed the voltage-dependent reductions and increases of the activation (*d*_L_) and inactivation gating variable (*f*_L_), respectively, in the L-type Ca^2+^ channel (LTCC) current (*I*_CaL_) (Supplementary Fig. S1A*b* and A*c*). The *I*_CaL_ was defined by the product of *d*_L_, *f*_L_ and the Ca^2+^-dependent inactivation (*f*_CaL_) state (Supplementary Fig. S1A*d*); hence the deactivation delay of the *d*_*L*_-gate caused an augmentation of the *I*_CaL_ window current during late AP phase 2 with each stimulation (section signs in Fig. 3G and Supplementary Fig. S1A*e*). Furthermore, the AP prolongation increased Ca^2+^ influx via the LTCC (Fig. 3F), and led to the elevation of [Ca^2+^]_i_ (Fig. 3H). The [Ca^2+^]_i_ elevation enhanced NCX activity, consequently leading to the large inward shift of *I*_NCX_ during AP phase 2 (Fig. 3E). The augmented *I*_CaL_ window current and large inward shift of *I*_NCX_ enhanced an inward current component in the net ionic current (*I*_net_) during late AP phase 2 (Supplementary Fig. S1A*f*). As the inward current components in *I*_net_ increased with each stimulation, the *I*_net_ temporarily caused a balance between the inward and outward currents (sharps in Supplementary Fig. S1A and S1B) that interrupted AP repolarisation. Then, the predominance of inward currents caused a transition from AP repolarisation to depolarisation during the AP phase 2. The initiation of transient depolarisation during the AP phase 2 in the EAD1 response coincided with the timing of the inward-outward balance of *I*_net_ and the reactivation of the *d*_L_ in *I*_CaL_ (red dashed line in Supplementary Fig. S1A and S1B). Once EAD emerged, Ca^2+^ influx via LTCC markedly increased (Fig. 3G and H), causing elevation of Ca^2+^ concentration in the network ([Ca^2+^]_NSR_) and junctional sarcoplasmic reticulum ([Ca^2+^]_JSR_) (Supplementary Fig. S1A*g* and A*h*). This in turn led to the elevation of [Ca^2+^]_i_ via increases in Ca^2+^ release from junctional sarcoplasmic reticulum to the myoplasm, i.e. Ca^2+^-induced Ca^2+^ release (CICR) (Supplementary Fig. S1A*i*), resulting in the elevation of transient [Ca^2+^]_i_ peaks (Fig. 3H).

### Modification of EAD formations by attenuation of the repolarisation reserve

**Figure 4:**
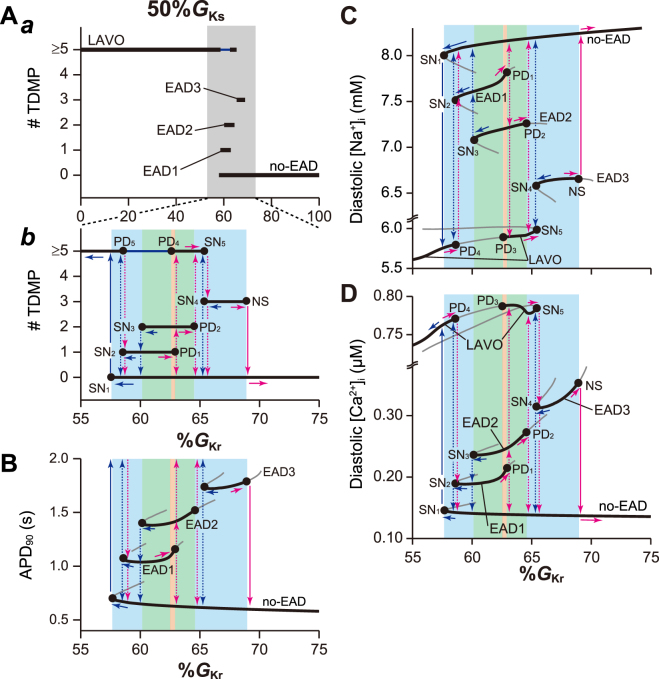
Effects of decreased *I*_Ks_ on the formation of early afterdepolarisations. One-parameter bifurcation diagrams of the number of transiently depolarised membrane potentials (#TDMP) during (**A**) action potential (AP) phase 2–3, (**B**) AP duration measured at 90% repolarisation, APD_90_, (**C**) diastolic [Na^+^]_i_, and (**D**) diastolic [Ca^2+^]_i_, as a function of the maximum conductance (%*G*_Kr_) of *I*_Kr_ with 50%*I*_Ks_. In A, panel *b* represents an enlargement of the grey %*G*_Kr_ region in panel *a*. The coloured %*G*_Kr_ regions indicate parameters at which bi-stable (cyan), tri-stable (green), and tetra-stable (orange) dynamics can be observed. Other symbols are as indicated in Fig. 2.

**Figure 5:**
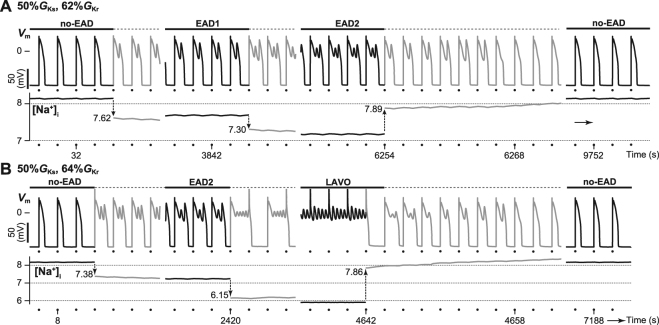
Representative examples of tri-stable action potential (AP) dynamics observed in the ventricular myocyte model. Simulated AP trains (top) and changes in intracellular Na^+^ concentration ([Na^+^]_i_) (bottom) at (**A**) 62%*G*_Kr_, and (**B**) 64%*G*_Kr_ with 50%*G*_Ks_. The [Na^+^]_i_ was further perturbed at appropriate times during the simulated AP trains, indicated by arrows with values in mM. Black and grey traces indicate the steady state and transient responses, respectively. Dots indicate the application of current pulses. Pacing cycle length = 2 s.

Next, we investigated the combined effects of decreases in both *I*_Kr_ and *I*_Ks_ on dynamical behaviours in the VM model. As *I*_Ks_ decreased, all bifurcation points gradually shifted toward higher %*G*_Kr_ values (Fig. 4 and Supplementary Fig. S2). The *I*_Ks_ reduction also resulted in a right shift of the %*G*_Kr_ range at which black solid lines overlapped in Fig. 2, which caused increasingly complicated multi-stability and hysteresis. The reason for the rightward shift of %*G*_Kr_, which causes EADs, can be intuitively understood as follows: the potential *I*_Ks_ decline leads to the AP prolongation, increasing the *I*_CaL_ window current and *I*_NCX_ inward shift during AP phase 2. These inward current increases tend to cause inward-outward current balance in the *I*_net_ during AP phase 2. Thus, the repolarization reserve attenuation accompanied by the decrease in *I*_Ks_ facilitates the EAD formation with the *I*_Kr_ reduction. Figure 5 shows representative examples of tri-stable dynamics that depended on initial conditions (Supplementary Table S3). The transitions among AP responses were caused by the dissipative or accumulative perturbations in [Na^+^]_i_. Reducing *I*_Kr_ along with the decrease in *I*_Ks_ formed a new low-amplitude voltage oscillation (LAVO), as shown in Fig. 5B. The LAVO was a *V*_m_ oscillation at near plateau potential, and caused repolarisation failure, i.e. arrest at the depolarised potential. Four distinct stable APs (no-EAD, EAD1, EAD2, and LAVO) coexisted at a range of %*G*_Kr_ (orange region in Fig. 4), indicating tetra-stable dynamics (Supplementary Fig. S3A). The VM model also exhibited tetra-stable dynamics (Supplementary Fig. S3B) at 70%*G*_Ks_ (orange region in Supplementary Fig. S2), which converged to either no-EAD, EAD1, EAD2 or EAD3 responses, depending on the initial condition (Supplementary Table S4).

The reduction of %*G*_Ks_ caused complicated transitions between attractors. In Fig. 4 and Supplementary Fig. S2, the vertical blue and magenta arrows show the relationship of AP response transitions that occur as a result of a bifurcation when the %*G*_Kr_ was reduced (horizontal blue arrows) or increased (horizontal magenta arrows). The annihilation of the no-EAD response caused by the SN_1_ bifurcation resulted in the transition to a LAVO response (see solid vertical blue arrow in Fig. 4), while the SN1 bifurcation of the no-EAD response in Supplementary Fig. S2 could transition to either EAD1, EAD2, or EAD3 responses (dashed vertical blue arrows), depending on the state variables just after the SN_1_ bifurcation. Furthermore, when the EAD1, EAD2, and EAD3 responses were annihilated by the occurrence of SN_2_, SN_3_, and SN_4_ bifurcations, respectively, the emerging AP response was not uniquely determined due to multi-stability (dashed vertical blue arrows in Fig. 4 and Supplementary Fig. S2). For example, the annihilation of the EAD1 response in Fig. 4 could potentially cause a transition to the no-EAD or LAVO response. In contrast, upon increasing %*G*_Kr_, the EAD1, EAD2, and EAD3 responses destabilised due to the occurrence of PD_1_, PD_2_, and Neimark-Sacker (NS) bifurcations, respectively. Furthermore, the PD_4_ or SN_5_ bifurcation destabilised or annihilated the LAVO response, respectively; for example, destabilisation of the EAD1 response causes a transition to either no-EAD, EAD2, or LAVO (dashed vertical magenta arrows in Fig. 4), and EAD2 destabilisation potentially transitions to a no-EAD or a LAVO response.

### Contribution of [Na^+^]_i_ and [Ca^2+^]_i_ to multi-stable dynamics

**Figure 6:**
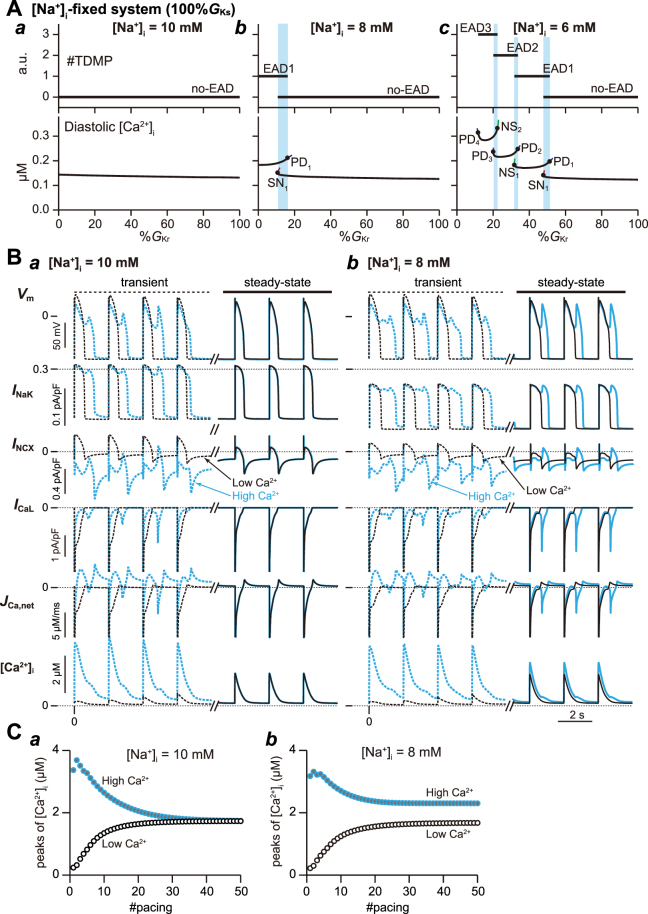
Contribution of [Na^+^]_i_- and [Ca^2+^]_i_-variability to the multi-stable dynamics. (**A**) One-parameter bifurcation diagrams of the number of transiently depolarised membrane potentials (#TDMP) during action potential (AP) phase 2–3 (top) and diastolic [Ca^2+^]_i_ (bottom) in the [Na^+^]_i_-fixed system as a function of %*G*_Kr_. The cyan region indicates the %*G*_Kr_ values at which the [Na^+^]_i_-fixed system exhibits bi-stability. Other symbols are as indicated in Fig. 2. (**B**) Simulated AP trains (*V*_m_), *I*_NaK_, *I*_NCX_, net Ca^2+^ flux (*J*_Ca,net_) and [Ca^2+^]_i_ change at 15%*G*_Kr_ when [Na^+^]_i_ was fixed at 10 mM (panel *a*) and 8 mM (panel *b*). Each result was obtained by starting from the low Ca^2+^ condition ([Ca^2+^]_JSR_ = [Ca^2+^]_NSR_ = 0.01 mM; black traces) or the high Ca^2+^ condition ([Ca^2+^]_JSR_ = [Ca^2+^]_NSR_ = 6 mM; cyan traces). Solid and dashed traces indicate the steady state and transient responses, respectively. (**C**) Changes in peak [Ca^2+^]_i_ at each pacing stimulus with [Na^+^]_i_ fixed at 10 mM (panel *a*) and 8 mM (panel *b*).

To evaluate the impact of [Na^+^]_i_ variability on multi-stable AP behaviour in the VM model, we performed additional bifurcation analyses in the non-autonomous [Na^+^]_i_-fixed system. Figure 6A shows one-parameter bifurcation diagrams of AP responses observed in the model with [Na^+^]_i_ fixed at several values. When [Na^+^]_i_ is fixed at 10 mM, neither multi-stable AP responses nor EAD formations occurred (Fig. 6A*a*). Reducing %*G*_Kr_ at relatively low [Na^+^]_i_ (fixed at 8 mM and 6 mM) caused AP with EADs and bi-stable behaviour, but multi-stable AP responses did not occur. This suggests that the emergence of higher order multi-stable states depended on [Na^+^]_i_ variation.

Next we conducted AP simulations to examine differences between the mono-stable ([Na^+^]_i_ = 10 mM) and bi-stable AP behaviours ([Na^+^]_i_ = 8 mM) observed in the [Na^+^]_i_-fixed system at the same *I*_Kr_ value (Fig. 6B). Except for [Ca^2+^]_JSR_ and [Ca^2+^]_NSR_, initial values were adopted from a set of steady-state values of the state variables, at which both 10 and 8 mM [Na^+^]_i_-fixed systems exhibited no-EAD response at 15%*G*_Kr_ (Supplementary Table S5). In both systems with low Ca^2+^ conditions ([Ca^2+^]_JSR_ = [Ca^2+^]_NSR_ = 0.01 mM), evoked APs were consistently no-EAD responses, including the transient response (black dashed *V*_m_ traces in Fig. 6B*a* and B*b*), until steady state was obtained (black solid *V*_m_ traces in Fig. 6B*a* and B*b*). However, the APD in the 8 mM [Na^+^]_i_-fixed system was longer than that of the 10 mM [Na^+^]_i_-fixed system (651 vs. 528 ms, respectively), even at the same *I*_Kr_ (black *V*_m_ traces in Fig. 6B*a* and B*b*). This was because the low [Na^+^]_i_ (8 mM) attenuated NaK activity (black *I*_NaK_ traces in Fig. 6B*a* and B*b*), decreased the outward current component of *I*_net_ and augmented forward-mode NCX activity during AP phase 2 (black *I*_NCX_ traces in Fig. 6B*a* and B*b*); the inward shift of *I*_NCX_ increased the inward current component of *I*_net_. The low Ca^2+^ condition simulates a Ca^2+^-depleted myocyte, where a large beat-to-beat Ca^2+^ influx (black *J*_Ca,net_ traces in Fig. 6B*a* and B*b*) leads to [Ca^2+^]_i_ accumulation. Likewise, the [Ca^2+^]_i_ peak increased with each stimulation and converged to a single value (Fig. 6C*a* and C*b*).

In contrast, the [Na^+^]_i_-fixed systems exhibited different responses in high Ca^2+^ conditions ([Ca^2+^]_JSR_ = [Ca^2+^]_NSR_ = 6 mM) depending on the [Na^+^]_i_-fixed value. The AP response in the 10 mM [Na^+^]_i_ -fixed system transiently exhibited an EAD1 response (dashed cyan *V*_m_ trace in Fig. 6B*a*) that converged to a no-EAD response (solid cyan *V*_m_ trace in Fig. 6B*a*). In contrast, the AP response in the 8 mM [Na^+^]_i_-fixed system converged to an EAD1 response (cyan *V*_m_ trace in Fig. 6B*b*). In high Ca^2+^ conditions, Ca^2+^ efflux became dominant at the earlier phase of pacing stimuli (cyan dashed *J*_Ca,net_ traces in Fig. 6B*a* and B*b*) because the high Ca^2+^ concentration in the myocyte augmented NCX activity (cyan dashed *I*_NCX_ traces in Fig. 6B*a* and B*b*). In addition, high *I*_NaK_ activity in the 10 mM [Na^+^]_i_-fixed system (*I*_NaK_ in Fig. 6B*a*) contributed to the increase in the outward current component of *I*_net_, leading to AP shortening and DI prolongation. This DI prolongation further increased Ca^2+^ efflux, causing a rapid decline in [Ca^2+^]_i_ peaks (Fig. 6C*a*). The low [Na^+^]_i_ (8 mM) diminished *I*_NaK_ (*I*_NaK_ in Fig. 6B*b*) and the high Ca^2+^ condition augmented forward-mode *I*_NCX_ activity (*I*_NCX_ in Fig. 6B*b*); consequently, AP was prolonged and the *I*_CaL_ window current was increased in late AP phase 2. Increases in these inward current components of *I*_net_ eventually led to transient depolarisation during AP phase 2. AP prolongation with emerging transient depolarisation increased Ca^2+^ influx via reactivated *I*_CaL_ (black *I*_CaL_ and *J*_Ca,net_ traces in Fig. 6B*b*); hence the [Ca^2+^]_i_ peaks converged to a relatively high value compared to the low [Ca^2+^]_i_ condition (Fig. 6C*b*). These results suggest that the [Na^+^]_i_ variability was involved in the emergence of higher order multi-stable AP responses, and that which steady-state response appeared in bi-stable AP dynamical behaviour was mainly determined by the NCX activity strongly influencing [Na^+^]_i_ and [Ca^2+^]_i_ states.

## Discussion

The major findings of the present study are as follows: (1) attenuating the repolarisation reserve (*I*_Kr_ and/or *I*_Ks_) caused prolongation of no-EAD responses, and the AP was annihilated without EAD formation via SN bifurcation. The EADs observed after the annihilation of AP emerged as a transition from the no-EAD response to a new stable state. (2) The destabilisation of AP responses that was mediated by the occurrence of PD and/or NS bifurcations caused the disappearance of APs with EADs during *I*_Kr_ recovery. (3) These transitions among attractors with hysteresis were accompanied by the discontinuous change in various states, including [Na^+^]_i_, [Ca^2+^]_i_ and APD, consequently creating the bi- and multi-stable states. (4) The development of multi-stable AP dynamics was strongly related to *I*_NaK_ and *I*_NCX_ changes, which were mediated by [Na^+^]_i_ variability, and [Ca^2+^]_i_ dynamics under the multi-stable AP behaviour contributed to EAD formation and APD variability. Such APD variability resulting from multi-stable AP dynamics in cardiomyocytes might be responsible for arrhythmias arising from repolarisation reserve attenuation.

The repolarisation reserve attenuation resulted in EAD responses with different numbers of transiently depolarised membrane potentials during AP phase 2–3 (Figs 1, 5 and Supplementary Fig. S3). The evoked EAD patterns resembled those of erythromycin induced EADs recorded from the mid-myocardial cells in the canine left ventricle^10^. The EAD formations in our VM model were not due to destabilisation of *V*_m_ during AP phase 2–3^10, 16^ following excessive APD prolongation in no-EAD response with repolarisation reserve attenuation. The EAD emergence was due to the transition to the potentially-existing AP with EADs as a result of the annihilation of no-EAD response; therefore, EADs appeared to occur suddenly during %*G*_Kr_ reduction. This shows that the AP with EADs is not directly linked to *V*_m_ destabilization in the no-EAD response via AP prolongation. Furthermore, we found that the presented VM model exhibited multi-stable AP dynamics with hysteretic loops (Fig. 2, 4 and Supplementary Fig. S2). In our previous study^22^, we confirmed the bi-stable AP behaviour was also observed in the more sophisticated human VM model proposed by O’Hara *et al*.^23^ (referred to ORd model). In rabbit VMs and the *in silico* model, Xie *et al*.^17^ have also observed intermittent spontaneous switching between AP responses with and without EADs that are similar to bi-stable AP behaviour with hysteresis, and they demonstrated that the [Na^+^]_i_ variation played important roles in the occurrence of bi-stable dynamics. Such a hysteretic response in myocytes should be validated experimentally, but not yet at the moment. If a fine control of the *I*_Kr_/*I*_Ks_ inhibition using drug concentration changes is technically feasible, it might demonstrate the presence of hysteretic dynamics in cardiomyocyte.

We showed that whether an AP with or without EAD were evoked under the bi-stable condition depended on the initial [Na^+^]_i_ state (Fig. 3). Once steady-state AP responses in higher-order multi-stable behaviour was achieved, it was necessary to apply a relative large [Na^+^]_i_ perturbation to cause state transitions among the different steady-state AP responses (Fig. 5 and Supplementary Fig. S3). Furthermore, the [Na^+^]_i_ clamp in the [Na^+^]_i_-fixed system eliminated multi-stable behaviour in AP dynamics (Fig. 6A); thus, a wide [Na^+^]_i_ dynamic range was required for APs with different APDs to coexist as multi-stable states. These results suggest that [Na^+^]_i_ variability, mediated via NaK and NCX dynamics, is the underlying mechanism responsible for multi-stable AP dynamics. The NaK and NCX activity are relevant for Na^+^ extrusion amount upon AP development, and for the amount of Na^+^ loading during DI, respectively. Furthermore, AP prolongation under periodic stimuli results in DI shortening. Thus, AP prolongation increases the amount of Na^+^ extrusion via *I*_NaK_, whereas DI shortening decreases the amount of Na^+^ loading via *I*_NCX_. This extrusion and loading of Na^+^ generates the variation in [Na^+^]_i_. These imply that multi-stable AP dynamics depends significantly on the *I*_NaK_ and *I*_NCX_ models.

The initiation mechanism for the transiently depolarised membrane potential during AP phase 2–3 in the AP with EAD in the present study is essentially the same *I*_CaL_-dependent process that has been suggested in many previous studies^24–28^. The low [Na^+^]_i_ led to AP prolongation that was mediated by *I*_NaK_ decrease, as shown in previous experimental^29^ and theoretical studies^30^. This AP prolongation caused [Ca^2+^]_i_ elevation due to increase in Ca^2+^ influx via LTCCs resulting from the *I*_CaL_ window current increase (Fig. 3G). Consequently, the [Ca^2+^]_i_ elevation augmented forward-mode NCX activity (Fig. 3E), which increases Ca^2+^ extrusion as well as Na^+^ loading. This led to further prolongation of APD in a positive feedback manner^17^, resulting in an increase in AP repolarization delay. As the *I*_CaL_ window current and inward *I*_NCX_ shift increased with the AP repolarization delay, the inward-outward balance in *I*_net_ was created, forming a transient depolarisation during AP phase 2 (Supplementary Fig. S1B). Based on this result, the suppression of inward *I*_NCX_ shift might prevent the occurrence of transient depolarisation during AP phase 2 (i.e., EAD formation). Bourgonje *et al*.^31^ showed that combined NCX and LTCC inhibition was effective against TdP in dogs with chronic atrioventricular blocks. Thus, our results provide evidence that suppressing NCX activity might reduce the risk of EAD-induced arrhythmias.

The multi-stable AP behaviours may have significant implications for EAD-related arrhythmias. In addition to the *I*_Kr_ inhibition in patients with LQTS type 1 (*I*_Ks_ decrease) and type 2 (*I*_Kr_ decrease)^32, 33^, the unintended *I*_Kr_ inhibition by some drugs^7, 10^ may augment the risk of EAD development (Figs 2 and 4). Moreover, the hysteresis dynamics implies that, once EADs are elicited by *I*_Kr_ inhibition, greater *I*_Kr_ recovery (i.e. more than when the EADs were initiated) is required for suppression of the arrhythmogenic response. In addition, the emergence of multi-stable dynamics (Fig. 5 and Supplementary Fig. S3) may augment the APD heterogeneity of each myocyte in the ventricle. These predictions are based on the dynamical mechanism of EAD development, and are limited to the single cell model. As demonstrated by previous studies^19, 34–36^, the relationship between EAD development in single cell and arrhythmogenicity in multi-cellular (tissue) models becomes more complicated due to the existence of electrotonic interaction (i.e. gap-junction coupling). Although the EAD development in mid-myocardial cell may augment the transmural (global) APD heterogeneity as they are much more vulnerable to EAD formation than the endocardial and epicardial myocytes^13, 37, 38^, the local APD heterogeneity in well-coupled myocytes in the intact heart may decrease due to the averaging effect resulting from the electrotonic interaction^34, 35^. In contrast, diminishing electrotonic interaction, for example with ageing-related fibrosis, is known to facilitate the EAD formations^36, 39^ and thereby enhance the local and global heterogeneity in AP repolarisation. This may create suitable conditions for reentry^10^ and lead to the generation of lethal arrhythmias such as TdP^7, 40^. Therefore, the effects of multi-stable AP behaviours on EAD-induced arrhythmias need to be studied in more realistic two- and three-dimensional ventricular models.

Using the relatively simple VM model was advantageous as we were able to utilize bifurcation analysis as a means to elucidate the dynamical mechanism of EAD emergences, but the incompleteness of the human VM model was a major limitation. For instance, our model lacked some ionic currents, such as late *I*_Na_, and the *I*_Ks_ amplitude was relatively large compared to the experimental data obtained for basal human VMs^12, 23^. Our previous study^22^, however, showed that many bifurcation phenomena (i.e., mathematical structures in dynamical AP response) observed in the ORd model^23^ without pacing were essentially same as those of the presented VM model. The similarity of bifurcation structures between those models implies that the major dynamical properties of AP responses in the physiologically relevant human VM model may be represented by the relatively simple AP model. Nevertheless, more complex human VM models^23, 41–43^ with refined formulas for *I*_Ks_ and other components may be useful for understanding the detailed physiological and pathophysiological processes that are responsible for multi-stable AP phenomena. However, many of these complex human VM models are high dimensional systems, and to our knowledge, bifurcation analyses in such large-scale non-autonomous systems have not yet been successful. Such bifurcation analysis is theoretically possible, but is also practically challenging due to the extreme difficulty in obtaining an analytical description of the variational equations required for the evaluation of dynamical stability changes of cardiac AP responses (Supplementary Methods). Thus, dynamical system approaches in complex VM models would be highly challenging, and more research will be necessary to elucidate the precise roles of multi-stable AP behaviours in EAD-induced arrhythmias.

## Methods

### Ventricular myocyte model

As in our previous study^22^, we used the mid-myocardial cell version of a human VM model proposed by Kurata *et al*.^44^ that could reproduce phase 2 EADs during the inhibition of either *I*_Kr_ or *I*_Ks_ (i.e. attenuating the repolarisation reserve). The membrane currents include, *I*_K1_, *I*_Kr_, *I*_Ks_, *I*_NaK_, *I*_CaL_, *I*_NCX_, 4-aminopyridine-sensitive transient outward current (*I*_to_), Na^+^ channel current (*I*_Na_), Ca^2+^ pump current (*I*_pCa_) and background Na^+^ (*I*_Nab_) and Ca^2+^ (*I*_Cab_) currents. Time-dependent changes in the membrane potential (*V*_m_) are given by the formula:

1 $$d{V}_{{\rm{m}}}/{\rm{dt}}={I}_{{\rm{stim}}}-({I}_{{\rm{CaL}}}+{I}_{{\rm{Kr}}}+{I}_{{\rm{Ks}}}+{I}_{{\rm{to}}}+{I}_{{\rm{Na}}}+{I}_{{\rm{K}}1}+{I}_{{\rm{Nab}}}+{I}_{{\rm{Cab}}}+{I}_{{\rm{NaK}}}+{I}_{{\rm{NCX}}}+{I}_{{\rm{pCa}}}),$$

where *I*_stim_ represents the stimulus current (in pA/pF). The dynamics in the internal concentrations of Ca^2+^ ([Ca^2+^]_i_), Na^+^ ([Na^+^]_i_) and K^+^ ([K^+^]_i_) are modelled as first-order differential equations including material balance expressions. Details on the expressions of the VM model have been described previously^22, 44^. The model was implemented in an XML-based Physiological Hierarchy Markup Language (PHML), which is available at http://physiodesigner.org as an open-access resource. The external concentrations of Ca^2+^ ([Ca^2+^]_o_), Na^+^ ([Na^+^]_o_) and K^+^ ([K^+^]_o_) were fixed at 2, 140 and 5.4 mM, respectively. Furthermore, the [K^+^]_i_ was fixed at 140 mM as per previous studies^22, 45^.

### Bifurcation and analyses

The AP responses in the VM model were elicited with 80 pA/pF, 1 ms current pulses delivered at 0.5 Hz. Such a system can be defined as a periodic non-autonomous system, and dynamical responses in this system become periodic oscillations. Based on dynamical system theory^20, 21^, the study of qualitative properties of periodic oscillations in non-autonomous systems can be reduced to a diffeomorphism known as a Poincare map. Bifurcation occurs when a qualitative property (i.e. dynamical stability) of periodic oscillations changes due to altered system parameter values, e.g. the maximum conductance (*g*_Kr_) of *I*_kr_. We performed numerical calculations to detect bifurcation points using the homemade C language program as previously described^46–48^. Details are provided in the Supplementary Methods. In briefly, bifurcation analysis proceeded as follows: first, we identified a stable AP response using numerical simulation. Numerical integration continued until the state variable values at each application of the stimulus converged to a steady-state value. The steady-state value was defined as the value when the difference between each sampled value of state variables obtained from *k*-th and (*k* + 1)-th APs in a simulated AP train was below 1 × 10^−8^. Next, we computed accurate state variable values of the steady-state AP using a shooting method (e.g. Newton’s method) by using the sampled state variable values obtained previously as the initial condition. The accurate state variable values were used to simultaneously compute the characteristic multipliers determining the dynamical stability of the AP response (see Supplementary Methods). Then, we changed the parameter value (e.g. *g*_Kr_) slightly, and executed Newton’s method again using the preceding result as the new initial condition. If Newton’s method converges, we can obtain new state variable values and characteristic multipliers for the changed parameter value. The shooting method was performed for every changes in system parameter values until bifurcations were detected with the assessment of the characteristic multipliers; this method is known as continuation^21^. The bifurcations that may arise in the paced VM model are saddle-node (SN), period-doubling (PD), and the Neimark-Sacker (NS) bifurcations^21^. At the SN bifurcation point, two periodic APs (e.g. stable and unstable AP responses) coalesce and annihilate. The PD and NS bifurcations cause the stability change in a periodic AP response. As a side effect, another AP response with a doubled period is generated around the AP that caused the PD bifurcation. In NS bifurcation, a quasi-periodic AP response may occur in the VM model. Detailed descriptions for each bifurcation are provided in the Supplementary Methods.

### Bifurcation diagrams

In the present study, we constructed one-parameter bifurcation diagrams of periodic AP responses for changes in the *g*_Kr_ of *I*_kr_. The maximum conductances of *I*_Ks_ and *I*_Kr_ per unit area were set to 0.0257 nS/pF and 0.00738 nS/pF, respectively^22^, which we defined as the control values (*g*_Ks_ and *g*_Kr_). Throughout the article, the maximum conductance of *I*_Ks_ and *I*_Kr_ was expressed as a percentage of the control values, i.e. %*G*_Ks_ and %*G*_Kr_, respectively. Each of the state variables could be plotted on the vertical axis in a one-parameter bifurcation diagram. In the present study, diastolic [Na^+^]_i_ and/or [Ca^2+^]_i_ values that were sampled during each application of the stimulus were plotted as a function of %*G*_Kr_. Furthermore, we depicted one-parameter bifurcation diagrams of the %*G*_Kr_ in relation to APD_90_ and the number of transiently depolarised membrane potentials during AP phase 2.

### Data availability

All data generated or analysed during this study are included in this published article (and its Supplementary Information files).

## Electronic supplementary material

Supplementary information
