# Supplementary material for: Hysteretic Dynamics of Multi-Stable Early Afterdepolarisations with Repolarisation Reserve Attenuation: A Potential Dynamical Mechanism for Cardiac Arrhythmias
Source: Sci Rep. 2017 Sep 7;7:10771. doi: 10.1038/s41598-017-11355-1 (PMC5589958; doi:10.1038/s41598-017-11355-1)
Supplement: Supplementary file 1 — Supplementary information [file 41598_2017_11355_MOESM1_ESM.pdf]

Supplementary Information

Hysteretic Dynamics of Multi-Stable Early Afterdepolarisations with Repolarisation Reserve Attenuation: A Potential Dynamical Mechanism for Cardiac Arrhythmias

Kunichika Tsumoto[1,*], Yasutaka Kurata[2], Kazuharu Furutani[1,3,4], and Yoshihisa Kurachi[1,3,*]

[1]Department of Pharmacology, Graduate school of Medicine, Osaka University, Suita, 565-0871, Japan

[2]Department of School of Medicine, Kanazawa Medical University, Ishikawa, 920-0293, Japan

[3]Global Center for Medical Engineering and Informatics, Osaka University, Suita, 565-0871, Japan

[4]Department of Physiology and Membrane Biology, University of California Davis, Davis, 95616, USA

Supplementary Table S1. Initial values for action potentials (AP) without early afterdepolarisations (no-EAD response) observed in the ventricular myocyte model with periodic stimuli.

**Variables**	Definitions	**100%G_{Ks}, 100%G_{Kr}**
V_m	Membrane potential	−85.83361
m	Activation gating variable for I_{Na}	1.34486×10^{-3}
h	Inactivation gating variable for I_{Na}	0.99406
d_L	Activation gating variable for I_{CaL}	4.23381×10^{-6}
f_L	Inactivation gating variable for I_{CaL}	0.99058
r	Activation gating variable for I_{to}	2.56525×10^{-5}
q	Inactivation gating variable for I_{to}	0.99956
P_a	Activation gating variable for I_{Kr}	1.19812×10^{-4}
n	Activation gating variable for I_{Ks}	0.01768
d_R	Activation gating variable for SR Ca^{2+} release channel	4.23389×10^{-6}
f_R	Inactivation gating variable for SR Ca^{2+} release channel	0.99986
f_{TC}	Fractional occupancy of the troponin by Ca^{2+}	0.20381
$[Ca^{2+}]_i$	Intracellular Ca^{2+} concentration	0.12797×10^{-3}
$[Ca^{2+}]_{JSR}$	Ca^{2+} concentration in the junctional SR	1.50785
$[Ca^{2+}]_{NSR}$	Ca^{2+} concentration in the network SR	1.53747
$[Na^+]_i$	Intracellular Na^+ concentration	8.79326

Supplementary Table S2. Initial values for action potentials (AP) without early afterdepolarisations (no-EAD response) for which bi-stable dynamics could be observed in the ventricular myocyte model.

**100%G_{Ks}, 12%G_{Kr}**	
**Variables**	no-EAD
V_m	−85.63930
m	1.38920×10^{-3}
h	0.99378
d_L	4.36600×10^{-6}
f_L	0.98606
r	2.60407×10^{-5}
q	0.99955
P_a	1.79209×10^{-4}
n	0.01784
d_R	4.36610×10^{-6}
f_R	0.99986
f_{TC}	0.22052
$[Ca^{2+}]_i$	0.14143×10^{-3}
$[Ca^{2+}]_{JSR}$	1.81069
$[Ca^{2+}]_{NSR}$	1.82204

Supplementary Table S3. Initial values for each action potential (AP) response where the ventricular myocyte model exhibited tri-stable dynamics consisting of no early afterdepolarisations (no-EAD), EAD1 and EAD2 response (62%G_{Kr} with 50%G_{Ks}); and no-EAD, EAD2 and low-amplitude voltage oscillation (LAVO) responses (64%G_{Kr} with 50%G_{Ks}).

	**50%G_{Ks}, 62%G_{Kr}**		
**Variables**	no-EAD	EAD1	EAD2
V_m	−85.65496	−85.19303	−84.74730
m	1.38557×10^{-3}	1.49653×10^{-3}	1.61186×10^{-3}
h	0.99380	0.99308	0.99230
d_L	4.35520×10^{-6}	4.68545×10^{-6}	5.02788×10^{-6}
f_L	0.98633	0.95178	0.87812
r	2.60092×10^{-5}	2.69547×10^{-5}	2.79000×10^{-5}
q	0.99955	0.99952	0.99948
P_a	1.70482×10^{-4}	1.48010×10^{-3}	1.16788×10^{-2}
n	0.01782	0.01843	0.02178
d_R	4.35529×10^{-6}	4.68567×10^{-6}	5.02865×10^{-6}
f_R	0.99986	0.99985	0.99984
f_{TC}	0.21823	0.28136	0.32459
$[Ca^{2+}]_i$	0.13956×10^{-3}	0.19572×10^{-3}	0.24019×10^{-3}
$[Ca^{2+}]_{JSR}$	1.77049	2.90152	3.62759
$[Ca^{2+}]_{NSR}$	1.78377	2.88777	3.61542
$[Na^{+}]_i$	8.14680	7.69469	7.18104

Supplementary Table S3. (*Continued*)

	**50%G_{Ks}, 64%G_{Kr}**		
**Variables**	no-EAD	EAD2	LAVO
V_m	−85.66612	−84.57922	1.74545
m	1.38299×10^{-3}	1.65757×10^{-3}	0.99568
h	0.99382	0.99198	1.27189×10^{-7}
d_L	4.34751×10^{-6}	5.16345×10^{-6}	0.81244
f_L	0.98665	0.84046	0.08291
r	2.59867×10^{-5}	2.82653×10^{-5}	4.42459×10^{-2}
q	0.99955	0.99946	3.77984×10^{-3}
P_a	1.65409×10^{-4}	2.15768×10^{-2}	0.74559
n	0.01782	0.02513	0.47287
d_R	4.34760×10^{-6}	5.16481×10^{-6}	0.79098
f_R	0.99986	0.99983	0.04117
f_{TC}	0.21712	0.34458	0.61131
$[Ca^{2+}]_i$	0.13865×10^{-3}	0.26269×10^{-3}	0.78689×10^{-3}
$[Ca^{2+}]_{JSR}$	1.75050	3.91315	5.88131
$[Ca^{2+}]_{NSR}$	1.76482	3.90608	5.90565
$[Na^+]_i$	8.17854	7.24603	5.90836

Supplementary Table S4. Initial values for each action potential (AP) response where the ventricular myocyte model exhibited tetra-stable dynamics consisting of no early afterdepolarisations (no-EAD), EAD1, EAD2 and low-amplitude voltage oscillation (LAVO) response (63%G_{Kr} with 50%G_{Ks}); or no-EAD, EAD1, EAD2 and EAD3 response (39%G_{Kr} with 70%G_{Ks}).

	**50%G_{Ks}, 63%G_{Kr}**			
**Variables**	no-EAD	EAD1	EAD2	LAVO
V_m	−85.66084	−85.06837	−84.69436	4.20551
m	1.38421×10^{-3}	1.52795×10^{-3}	1.62612×10^{-3}	0.99671
h	0.99381	0.99287	0.99220	6.95740×10^{-8}
d_L	4.35114×10^{-6}	4.77880×10^{-6}	5.07019×10^{-6}	0.86625
f_L	0.98650	0.93339	0.86647	0.06709
r	2.59974×10^{-5}	2.72159×10^{-5}	2.80146×10^{-5}	6.51667×10^{-2}
q	0.99955	0.99951	0.99947	5.47638×10^{-3}
P_a	1.67762×10^{-4}	3.00690×10^{-3}	1.43390×10^{-2}	0.76594
n	0.01782	0.01888	0.02265	0.47920
d_R	4.35124×10^{-6}	4.77916×10^{-6}	5.07112×10^{-6}	0.85817
f_R	0.99986	0.99984	0.99983	0.02386
f_{TC}	0.21764	0.30210	0.33137	0.61182
$[Ca^{2+}]_i$	0.13907×10^{-3}	0.21637×10^{-3}	0.24767×10^{-3}	0.78841×10^{-3}
$[Ca^{2+}]_{JSR}$	1.75986	3.26115	3.72987	5.88220
$[Ca^{2+}]_{NSR}$	1.77369	3.24700	3.71895	5.90357
$[Na^+]_i$	8.16330	7.82910	7.21313	5.89683

Supplementary Table S4. (*Continued*)

	**70%G_{Ks}, 39%G_{Kr}**			
**Variables**	No-EAD	EAD1	EAD2	EAD3
V_m	−85.62412	−85.26516	−84.69288	−84.07248
m	1.39272×10^{-3}	1.47865×10^{-3}	1.62652×10^{-3}	1.80325×10^{-3}
h	0.99376	0.99320	0.99220	0.99094
d_L	4.37650×10^{-6}	4.63228×10^{-6}	5.07139×10^{-6}	5.59480×10^{-6}
f_L	0.98549	0.95983	0.86630	0.71558
r	2.60713×10^{-5}	2.68049×10^{-5}	2.80178×10^{-5}	2.93963×10^{-5}
q	0.99955	0.99952	0.99947	0.99942
P_a	1.87128×10^{-4}	1.00400×10^{-3}	1.43607×10^{-2}	7.97584×10^{-2}
n	0.01785	0.01828	0.02257	0.05055
d_R	4.37659×10^{-6}	4.63246×10^{-6}	5.07240×10^{-6}	5.59889×10^{-6}
f_R	0.99986	0.99984	0.99983	0.99981
f_{TC}	0.22186	0.27127	0.33510	0.38258
$[Ca^{2+}]_i$	0.14253×10^{-3}	0.18609×10^{-3}	0.25185×10^{-3}	0.30939×10^{-3}
$[Ca^{2+}]_{JSR}$	1.83503	2.72119	3.78571	4.32634
$[Ca^{2+}]_{NSR}$	1.84522	2.70900	3.77557	4.33073
$[Na^{+}]_i$	8.07775	7.71236	7.36295	6.80124

Supplemental Table S5. Initial values for the no early afterdepolarisation (no-EAD) response observed in the 10 and 8 mM $[Na^+]_i$-fixed systems.

**100%G_{Ks}, 15%G_{Kr}**		
**Variables**	10 mM $[Na^+]_i$-fixed system	8 mM $[Na^+]_i$-fixed system
V_m	−85.95256	−85.62547
m	1.31841×10^{-3}	1.39241×10^{-3}
h	0.99423	0.99376
d_L	4.15487×10^{-6}	4.37556×10^{-6}
f_L	0.98956	0.98620
r	2.54177×10^{-5}	2.60685×10^{-5}
q	0.99957	0.99954
P_a	1.28148×10^{-4}	1.76751×10^{-4}
n	0.01759	0.01785
d_R	4.15494×10^{-6}	4.37566×10^{-6}
f_R	0.99986	0.99985
f_{TC}	0.21985	0.21892
$[Ca^{2+}]_i$	0.14088×10^{-3}	0.14012×10^{-3}

Supplementary Methods for Numerical Bifurcation Analyses

A composite dynamical system

Action potential (AP) responses in a ventricular myocyte model shown in Equation 1 are evoked by periodic external current stimuli (I_{stim}). The temporal changes in I_{stim} are expressed as:

$$I_{\text{stim}}(t) = \begin{cases} I_{\text{stim,max}} & (0 \le t < T_d) \\ 0 & (T_d \le t < T) \end{cases}, \qquad \text{(S1)}$$

where T is the pacing cycle length (i.e. period of the current stimuli) and T_d is the duration for which the stimulus current is sustained at the maximum value ($I_{\text{stim,max}}$). The first and second equations in Equation S1 corresponds to the current injection phase and the no-injection phase of the external current stimulus, respectively. During the current injection phase and the no-injection phase, the ventricular myocyte (VM) model becomes an autonomous system with constant parameters $I_{\text{stim,max}}$ and zero. Therefore, the paced VM model can be formalised as a composite dynamical system such that the two corresponding autonomous systems are successively switched over time.

Poincaré map of the composite dynamical system

To investigate stability changes in periodic oscillations when a system parameter is changed, we directly assessed the dynamical stability of AP responses using a method involving the Poincaré map. Even though the VM model driven by a discontinuous periodic force (Eq. S1) had a discontinuous nature, the Poincare map could be constructed numerically as successive submaps[1,2].

Consider the following non-autonomous differential equations consisting of Equation 1 and a periodic parameter variation of Equation S1 during the time satisfying $t - t_0 \pmod{T} \in [0,T)$:

$$\frac{\mathrm{d}x}{\mathrm{d}t} = f(t,x,\lambda) = \begin{cases} f_1(x,\lambda_0,\lambda_a) & (0 \le t < T_d) \\ f_2(x,\lambda_0) & (T_d \le t < T) \end{cases}, \qquad \text{(S2)}$$

where $t \in R$ denotes time, $x \in R^n$ is the state vector, $\lambda_0 \in R^{m-1}$ denotes common parameters for f and $\lambda_a \in \mathrm{R}$ is a parameter specifying f_1. The parameters λ_a correspond to $I_{stim,max}$. We also assumed that f is periodic in T so that $f(t + T, x, \lambda) = f(t, x, \lambda)$, for all t. If we assumed that the solution to Equation S2 is described as a mixed solution of the first and second equations of Equation S2, then the solution with the initial condition $x = x_0$ at $t = t_0$ is represented by:

$$x(t) = \varphi(t,\lambda;t_0,x_0) = \varphi(t,\lambda_0,\lambda_a;t_0,x_0). \qquad \text{(S3)}$$

Let φ_1 and φ_2 correspond to solutions to the first and second equations of Equation S2

$$x_1(t) = \varphi_1(t,\lambda_0,\lambda_a;t_0,x_0), \quad (t_0 \le t < t_0 + T_d),$$

and

$$x_2(t) = \varphi_2(t,\lambda_0;t_0+T_d,\varphi_1(t_0+T_d,\lambda_0,\lambda_a;t_0,x_0)), \quad (t_0+T_d \le t < t_0+T).$$

Then, the Poincaré map can be defined as a composite map:

$$\begin{aligned} M &: R^n \to R^n \\ & x_0 \mapsto M(x_0) = \varphi(t_0+T,\lambda_0,\lambda_a;t_0,x_0) \end{aligned} \qquad \text{(S4)}$$

and $M = M_2 \circ M_1$, to avoid discontinuity in the derivative of the solution at $t = t_0$ and $t = t_0 + T_d$. M_1 and M_2 are given by the sub-maps:

$$\begin{aligned} M_1 &: R^n \to R^n \\ & x_0 \mapsto x_1 = \varphi_1(t_0+T_d,\lambda_0,\lambda_a;t_0,x_0), \end{aligned}$$

and

$$\begin{aligned} M_2 &: R^n \to R^n \\ & x_1 \mapsto x_2 = \varphi_2(t_0+T,\lambda_0;t_0+T_d,x_1). \end{aligned}$$

Thereby, the study of a periodic oscillation becomes topologically equivalent to that of a fixed point on the Poincaré map.

Numerical calculation of the fixed point and detection of bifurcation points

We defined a fixed-point equation based on the fixed point on the Poincaré map (Eq. S4):

$$H(x_0) := x_0 - M(x_0) = 0, \qquad \text{(S5)}$$

where $x_0 \in R^n$ denotes the initial value at $t = t_0$. If an initial value (x_0) satisfies Equation S5, then this point is denoted a fixed point. We can also define the characteristic equation of the fixed point as follows:

$$\det\left[\mu I_n - \frac{\partial M(x_0)}{\partial x_0}\right] = 0, \qquad \text{(S6)}$$

where I_n is the $n \times n$ identity matrix, and $\partial M(x_0)/\partial x_0$ denotes the derivative of M with respect to x_0. The solutions of Equation S6 provide the characteristic multipliers ($\mu \in R^n$) for the fixed point. The co-dimension one bifurcations produced by the VM model are saddle-node, period-doubling and Neimark-Sacker bifurcations[3-6]. The saddle-node bifurcation causes two periodic solutions to emerge or disappear. At a particular parameter value, one of the multipliers of Equation S6 satisfies the condition $\mu = 1$[3,4]. The period-doubling bifurcation occurs when one of the characteristic multiplier becomes $\mu = -1$, after which the stability of periodic oscillation changes. As a side effect, another oscillation with a doubled period is generated around the periodic solution[3,5]. The Neimark-Sacker bifurcation causes the stable periodic oscillation to destabilise, and a double-period oscillation (quasi-periodic solution) may occur in the original non-autonomous system. This type of bifurcation occurs when $|\mu| = 1$[3,5,6].

The fixed point equation of Equation S5 cannot be analytically solved; therefore, we used Newton's method, which is a numerical approach to computation. In the following, we assume that the initial value in Equation S2 is given by x_0, and $x_0^{(k)}$ is a first-guess of the fixed point. The recurrent formula for Newton's method is given by:

$$\begin{cases} x_0^{(k+1)} = x_0^{(k)} + \delta \\ DH\left(x_0^{(k)}\right)\delta + H\left(x_0^{(k)}\right) = 0 \end{cases}, \quad k = 0,1,2,\cdots, \qquad \text{(S7)}$$

where δ is the correction term, and $DH(x_0^{(k)})$ is the Jacobian matrix with respect to the initial value x_0, denoted by:

$$DH(x_0) = I_n - \frac{\partial M(x_0)}{\partial x_0}. \qquad \text{(S8)}$$

Then, the derivative of M with regard to x_0 is expressed by:

$$\frac{\partial M(x_0)}{\partial x_0} = \frac{\partial \varphi(t_0 + T, \lambda_0, \lambda_a; t_0, x_0)}{\partial x_0}. \qquad \text{(S9)}$$

The second equation of Equation S7 must be solved for δ, using a suitable method such as Gauss elimination. $H(x_0^{(k)})$ can be obtained from the original Equation S5. Consequently, to obtain each element of the Jacobian matrix, we need to differentiate M with respect to x_0.

We defined the solution starting from x_0 at $t = t_0$ as:

$$x(t) = \varphi(t, \lambda; t_0, x_0) \equiv \varphi(t, x_0). \qquad \text{(S10)}$$

Substituting the solution of this equation into Equation S2, we get:

$$\frac{\mathrm{d}\varphi(t, x_0)}{\mathrm{d}t} = f(t, \varphi(t, x_0)). \qquad \text{(S11)}$$

Differentiating this equation by x_0 yields:

$$\frac{\partial}{\partial x_0}\left(\frac{\mathrm{d}\varphi(t, x_0)}{\mathrm{d}t}\right) = \frac{\partial f(t, \varphi(t, x_0))}{\partial x_0}. \qquad \text{(S12)}$$

The order of differentiation on the left-hand side is commutative, and the following equation is obtained from the right-hand side:

$$\frac{\mathrm{d}}{\mathrm{d}t}\left(\frac{\partial \varphi(t, x_0)}{\partial x_0}\right) = \frac{\partial f(t, \varphi(t, x_0))}{\partial x} \times \frac{\partial \varphi(t, x_0)}{\partial x_0}. \qquad \text{(S13)}$$

This equation is of the following form:

$$\frac{\mathrm{d}Y}{\mathrm{d}t} = \frac{\partial f(t, \varphi(t, x_0))}{\partial x} \times Y,$$

where $Y \equiv \partial\varphi/\partial x_0$ is the matrix solution of a variable coefficient linear differential equation,

referred to as a *variational equation* for Equation S2. Then, from Equation S10:

$$\frac{\partial \varphi(t_0, x_0)}{\partial x_0} = I \, . \qquad \text{(S14)}$$

Therefore, we can use Equation S14 as the initial value to calculate the derivative of M with regard to x_0, by numerically integrating Equation S13 from $t = t_0$ to $t_0 + T$. The Runge-Kutta method was used, after which Newton's method was performed. The fixed point x_0 is accurately located by iteration. However, the derivatives $\partial\varphi/\partial x_0$ cannot be directly defined due to discontinuities in the derivative of the solution. To avoid the impossibility of the derivative at $t = t_0 + T_d$ and $t_0 + T$, the first derivative of M with respect to x_0 is given by obtaining the derivatives of the sub-maps, successively:

$$\begin{aligned}\frac{\partial M(x_0)}{\partial x_0} &= \frac{\partial M_2(x_1)}{\partial x_1}\frac{\partial M_1(x_0)}{\partial x_0} \\ &= \frac{\partial \varphi_2(t_0+T, \lambda_0, \lambda_a; t_0+T_d, x_1)}{\partial x_1} \times \frac{\partial \varphi_1(t_0+T_d, \lambda_0, \lambda_a; t_0, x_0)}{\partial x_0} .\end{aligned} \qquad \text{(S15)}$$

In Equation S6, the derivatives of φ_i, for i = 1, 2 relate to the initial value x_k for k = 0, 1, which corresponds to fundamental matrix solutions (i.e. $\partial\varphi_i/\partial x_k$), and can be obtained by numerically integrating each of the first-order variational equations:

$$\frac{\mathrm{d}}{\mathrm{d}t}\left(\frac{\partial \varphi_1}{\partial x_0}\right) = \frac{\partial f_1}{\partial x}\frac{\partial \varphi_1}{\partial x_0} \qquad \text{with} \quad \left.\frac{\partial \varphi_1}{\partial x_0}\right|_{t=t_0} = I \, , \qquad \text{(S16)}$$

and

$$\frac{d}{dt}\left(\frac{\partial \varphi_2}{\partial x_1}\right) = \frac{\partial f_2}{\partial x}\frac{\partial \varphi_2}{\partial x_1} \qquad \text{with} \quad \left.\frac{\partial \varphi_2}{\partial x_1}\right|_{t=t_0+T_d} = I \, , \qquad \text{(S17)}$$

and substituting $t = t_0 + T_d$ and $t_0 + T$ in the respective solutions to Equation S7 and S8.

Supplementary References

1. Tsumoto, K., Yoshinaga, T., Iida, H., Kawakami, H., & Aihara, K. Bifurcations in a mathematical model for circadian oscillations of clock genes. *J Theor Biol*. **239**(1), 101–122 (2006).
2. Tsumoto, K., Ueta, T., Yoshinaga, T., & Kawakami, H. Bifurcation analyses of nonlinear dynamical systems: From theory to numerical computations. *Nonlinear Theory and Its Applications, IEICE*. **3**(4), 458–476; 10.1587/nolta.3.458 (2012).
3. Kuznetsov, Y. A. Elements of applied bifurcation theory (3rd Ed). 632 (Springer-Verlag, New York, 2004).
4. Arnold, V. I. Geometrical methods in the theory of ordinary differential equations (2nd Ed) (ed. Levi, M.) 351 (Springer-Verlag, New York, 1988).
5. Guckenheimer, J., & Holmes, P. Nonlinear oscillations, dynamical systems, and bifurcations of vector fields. 462 (Springer-Verlag, New York, 1983).
6. Hale, J. K. & Koçak, H. Dynamics and bifurcation, 568 (Springer-Verlag, New York, 1991).

Supplementary Figure Legends

Figure S1: Mechanism of early afterdepolarisation (EAD). (A*a*) Simulated action potential (AP) change, (A*b*) d_L, (A*c*) f_L and (A*d*) f_{CaL} states in the I_{CaL}, (A*e*) I_{CaL}, (A*f*) net ionic current, I_{net}, (A*g*) $[Ca^{2+}]_{NSR}$, (A*h*) $[Ca^{2+}]_{JSR}$ and (A*i*) total Ca^{2+} release from junctional sarcoplasmic reticulum to myoplasm via ryanodine receptors during the development of bi-stable dynamics as shown in Figure 3. The cyan and black traces indicate no-EAD and EAD1 responses at 12%G_{Kr}, respectively. (B) Enlargements of the area indicated by the box in panel A. a.u., arbitrary unit.

Figure S2: Bifurcation point shifts and multi-stable state developments with decreasing in I_{Ks}. One-parameter bifurcation diagrams of the number of transiently depolarised membrane potentials (#TDMP) during (A) action potential (AP) phase 2-3, (B) APD_{90}, (C) diastolic $[Na^+]_i$ and (D) diastolic $[Ca^{2+}]_i$, as a function of the maximum conductance (%G_{Kr}) of I_{Kr} with 70%I_{Ks}. In A, panel *b* shows an enlargement of %G_{Kr} indicated in grey in panel *a*. Thick black and thin grey lines represent the parameter values at which stable and unstable periodic AP responses can be observed in the paced ventricular myocyte model, respectively. The %G_{Kr} ranges shown by cyan and green indicate the ranges at which the VM model exhibits bi- and tri-stable AP dynamics, respectively. SN, saddle-node bifurcation; PD, period-doubling bifurcation; NS, Neimark-Sacker bifurcation.

Figure S3: Examples of tetra-stable action potential (AP) dynamics observed in the ventricular myocyte model. Simulated AP trains (top) and changes in $[Na^+]_i$ (bottom) at (A) 63%G_{Kr} with 50%G_{Ks} and (B) 39%G_{Kr} with 70%G_{Ks}. The $[Na^+]_i$ was further perturbed at appropriate times, indicated by arrows with values in mM, during the simulated AP trains. Black and grey lines indicate the steady-state and transient responses, respectively. Dots indicate the application of current pulses. Pacing cycle length = 2 s.

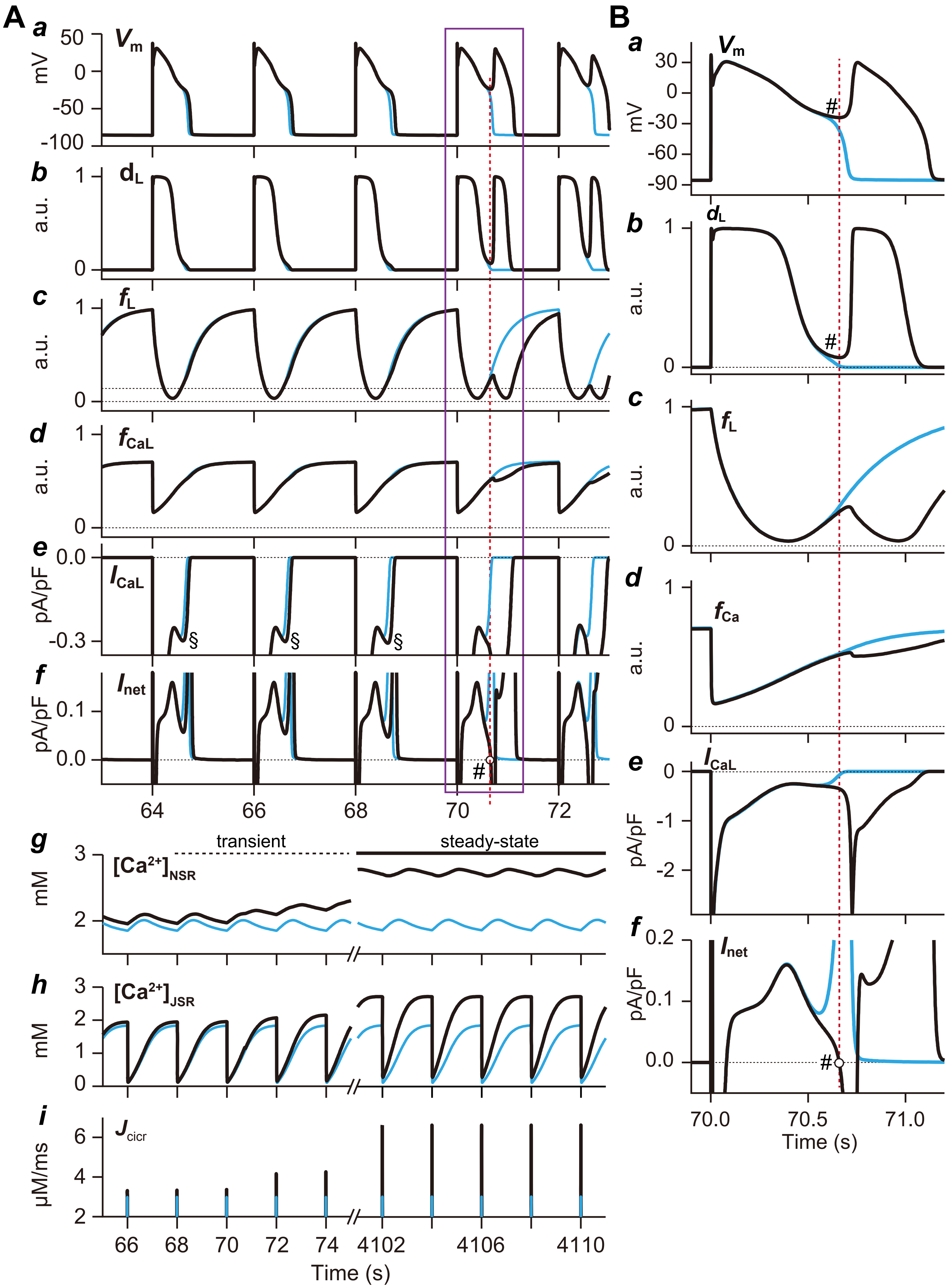

Supplementary Figure S1

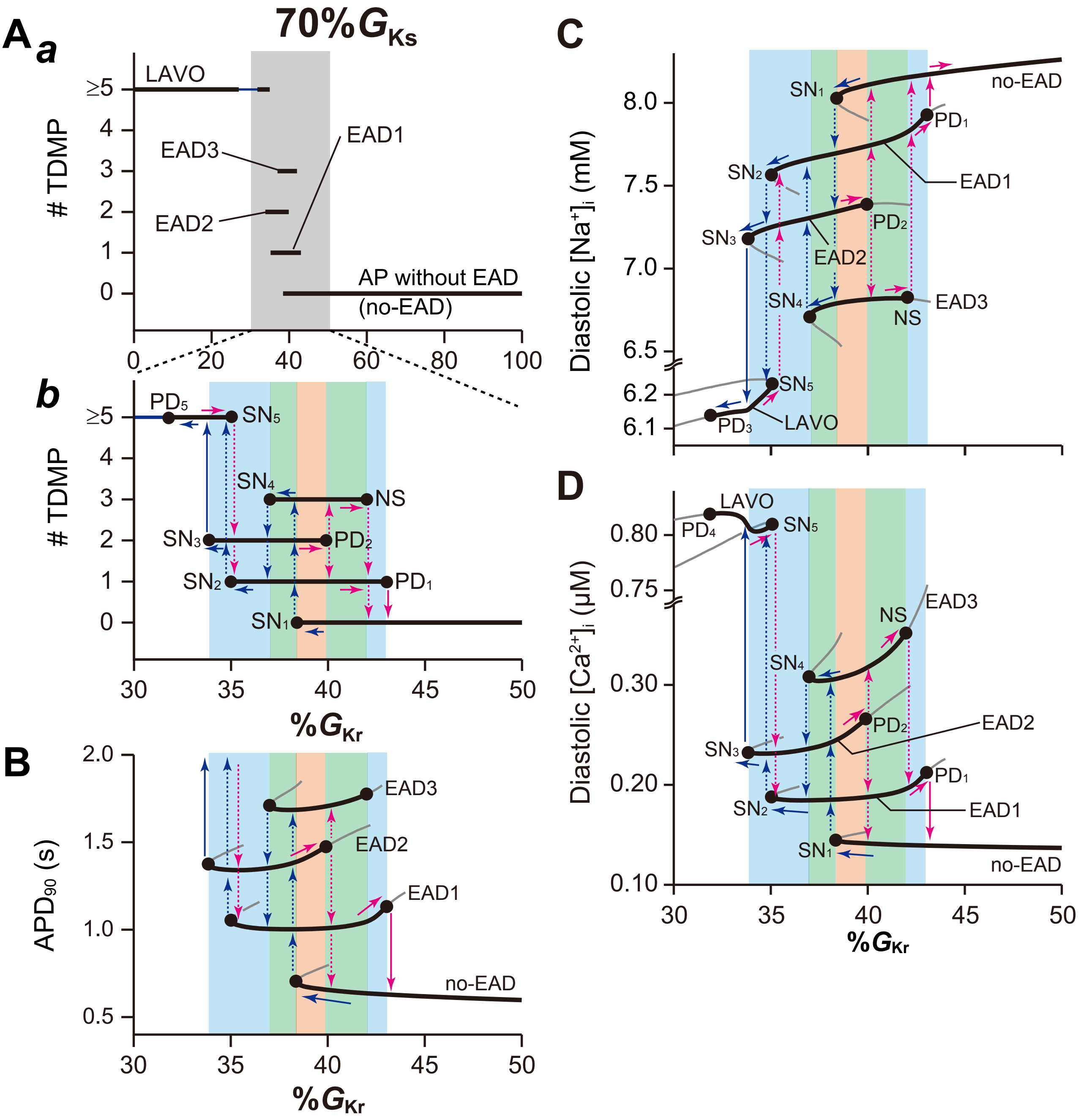

Suppplementary Figure S2

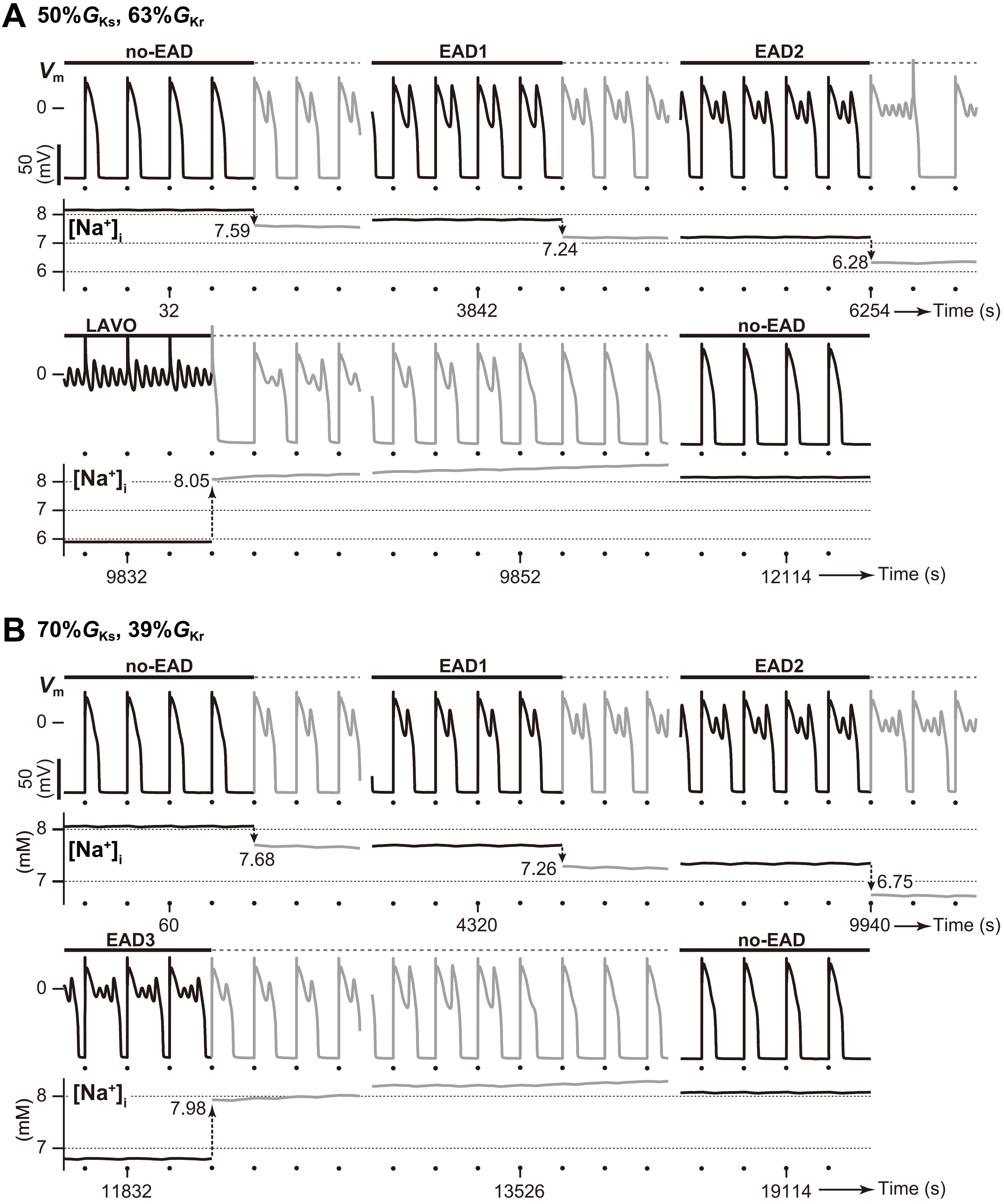

Supplementary Figure S3
